# Active Pedicle Epithelial Flap Transposition Combined with Amniotic Membrane Transplantation for Treatment of Nonhealing Corneal Ulcers

**DOI:** 10.1155/2016/5742346

**Published:** 2016-10-17

**Authors:** Ting Zhang, Yuexin Wang, Yanni Jia, Dongle Liu, Suxia Li, Weiyun Shi, Hua Gao

**Affiliations:** ^1^Qingdao University, 308 Ningxia Road, Qingdao 266071, China; ^2^Shandong Eye Hospital, Shandong Eye Institute, Shandong Academy of Medical Sciences, 372 Jingsi Road, Jinan 250021, China

## Abstract

*Introduction*. The objective was to evaluate the efficacy of active pedicle epithelial flap transposition combined with amniotic membrane transplantation (AMT) in treating nonhealing corneal ulcers.* Material and Methods*. Eleven patients (11 eyes) with nonhealing corneal ulcer who underwent the combined surgery were included. Postoperatively, ulcer healing time was detected by corneal fluorescein staining. Visual acuity, intraocular pressure, surgical complications, and recurrence were recorded. Corneal status was inspected by the laser scanning confocal microscopy and anterior segment optical coherence tomography (AS-OCT).* Results*. The primary diseases were herpes simplex keratitis (8 eyes), corneal graft ulcer (2 eyes), and Stevens-Johnson syndrome (1 eye). All epithelial flaps were intact following surgery, without shedding or displacement. Mean ulcer healing time was 10.8 ± 3.1 days, with a healing rate of 91%. Vision significantly improved from 1.70 to 0.82 log MAR (*P* = 0.001). A significant decrease in inflammatory cell infiltration and corneal stromal edema was revealed 2 months postoperatively by confocal microscopy and AS-OCT. Corneal ulcer recurred in 1 eye. None of the patients developed major complications.* Conclusion*. Active pedicle epithelial flap transposition combined with AMT is a simple and effective treatment for nonhealing corneal ulcers.

## 1. Introduction

A nonhealing corneal ulcer is defined as an ulcer which does not show any indication of healing within two weeks, despite the administration of proper medical treatment [1]. Suspicious causes of a nonhealing corneal ulcer include persistent infection, neurotrophic keratopathy, exposure keratopathy, dry eye, treatment toxicity, steroid use, and chronic conjunctival inflammation, such as ocular cicatricial pemphigoid [2]. Once a corneal ulcer occurs and is left unattended, corneal melting, descemetocele, and corneal perforation can subsequently develop, leading to devastating consequences [3]. Moreover, when a resistant corneal ulcer progressively develops, lamellar keratoplasty or penetrating keratoplasty is usually needed [4, 5]. Therefore, curing a resistant ulcer in its early stages is highly recommended.

Amniotic membrane transplantation (AMT) can be used to treat a superficial corneal ulcer. However, the efficacy of AMT, or even repeated AMT, in treating a nonhealing corneal ulcer is limited because the latter is usually associated with severe inflammatory response, which frequently causes corneal melting, earlier amniotic membrane dissolution, and deferred ocular surface epithelialization. Thus, reducing inflammatory cell infiltration and improving the local microenvironment may facilitate the healing of a resistant corneal ulcer.

Inspired by the significant anti-inflammation effects of the epithelial cells and the successful clinical application of laser-assisted subepithelial keratectomy (LASEK), which makes an epithelial flap with alcohol and significantly prevents haze formation in the subepithelial area [6], we attempted to treat nonhealing corneal ulcers in this study with the use of active pedicle epithelial flap transposition combined with AMT.

## 2. Material and Methods

### 2.1. Patients

This study was approved by the Institutional Review Board of Shandong Eye Institute, Qingdao, China, and conformed to the guidelines of the Declaration of Helsinki. Patients provided informed consent to participate in the study. The medical records of patients who had undergone active pedicle epithelial flap transposition combined with AMT for a nonhealing corneal ulcer between 1 March 2012 and 1 July 2015 were reviewed.

All of the patients had a history of eye hyperemia associated with pain and decreased vision. The ulcers were from 2 to 5 mm in diameter and less than 1/3 of the corneal stroma in depth. All ulcers were associated with corneal stroma melting and remained unhealed for ≥4 weeks despite the administration of medical treatment. The study mainly focused on nonhealing sterile corneal ulcers, and all patients received corneal scraping, culture, and laser scanning confocal microscopy examination to exclude active infection, for example, a fungal, resistant bacterial, or* Acanthamoeba* corneal ulcer.

### 2.2. Surgical Technique

**Figure 1:**
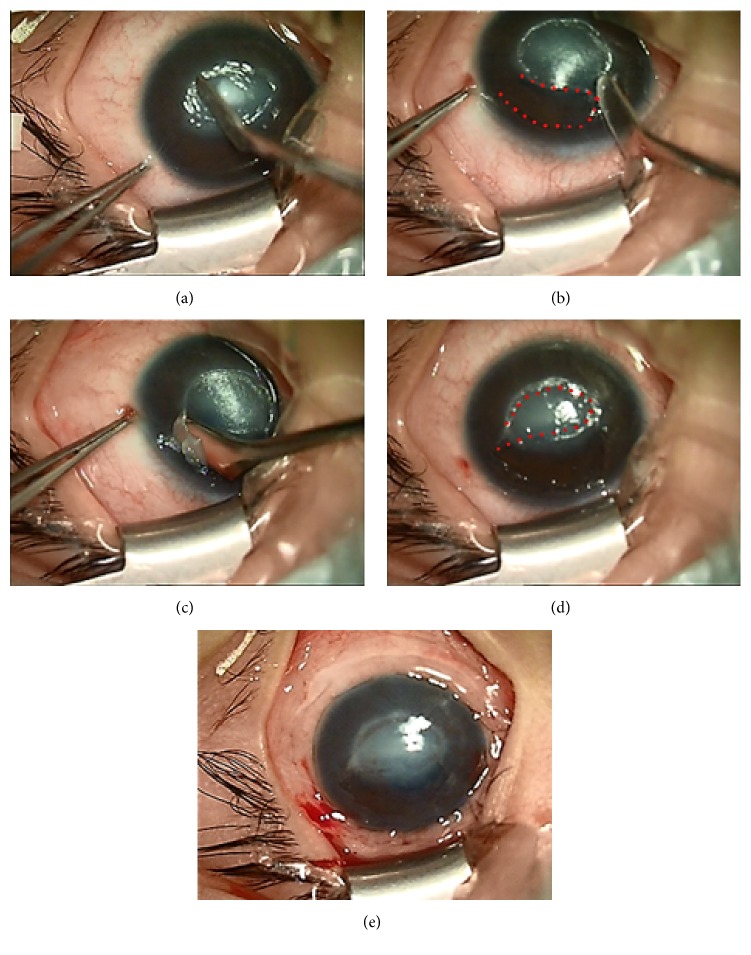
Photographs showing the procedures of epithelial flap transposition combined with amniotic membrane transplantation. (a) Cleaning the ulcer surface. (b) Constructing a flap boundary (the dotted line demonstrates the region from where the epithelial flap was obtained). (c) Obtaining an epithelial flap. (d) Transposing the epithelial flap (the dotted line demonstrates the translocated epithelial flap). (e) Suturing an amniotic membrane patch.

The amniotic membrane (AM) was prepared and preserved as previously reported [7], with minor modifications. The surgery was performed by the same surgeon. Necrotic corneal tissue was removed from the base and wall of the ulceration, and then thermal cautery was applied to the ulcer to dry out its surface. Thereafter, an epithelial flap was constructed from the transparent cornea at the edge of the ulcer, near the limbus. For this procedure, obtaining a flap from the edge of ulcer ensures easy transposition, and taking a flap from the cornea near the limbus guarantees rapid healing of the epithelial defect resulting from making a flap. In addition, a corneal epithelial scraper was used to create an epithelial flap without the use of alcohol to maintain epithelial cells activity. Briefly, the epithelial scraper was gently pressed onto the corneal surface to make a boundary of the flap shaped like the ulcer under the auxiliary arm of a caliper. A pedicle, like a sprout, was preserved in the lateral or nasal portion of the epithelial flap. The corneal epithelium was then carved following the boundary, with the depth confined to the epithelial layer. In this way, an epithelial flap was created by the corneal epithelial scraper. Next, the flap was rotated to cover the epithelial nonhealing region of the ulcer. To maintain flap adhesion, fluid on the ocular surface was cleaned with a sponge swab before covering it. Finally, a trephine was used to make an amniotic membrane of 14 mm in diameter, and the AM patch was sutured onto the surface to cover the entire cornea using a continuous 10-0 nylon suture within 1 mm of the limbus (Figure 1).

### 2.3. Postoperative Treatment

The primary diseases of nonhealing corneal ulcer in the study included herpes simplex keratitis (HSK), corneal graft ulcer, and Stevens-Johnson syndrome (SJS). Postoperatively, tobramycin and dexamethasone eye drops (Alcon, Puurs, Belgium) were used 4 times daily for 1-2 weeks and then replaced with 0.1% fluorometholone eye drops (Santen, Osaka, Japan) 4 times daily for approximately 1-2 months. For eyes with HSK, antiviral medication was applied topically and systemically with adjuvant corticosteroid eye drops [8, 9]. Acyclovir eye drops (Wuhan Wujing Pharmaceutical Co., Wuhan, China) were used 4 times every day. Ganciclovir ophthalmic gel (Hubei Keyi Pharmaceutical Co., Wuhan, China) was used every night. Acyclovir was administered orally (8 mg/kg) 3 times daily for 3 months. For eyes with prior corneal transplantation or Stevens-Johnson syndrome, 1% cyclosporine eye drops (Huabei Pharmaceutical Co., Shijiazhuang, China) were used 1–4 times daily.

The medical treatment was adjusted with the alleviation of symptoms and the dissolution of AM. Corneal fluorescein staining was performed daily and the sutures were removed 1–3 weeks postoperatively when the AM dissolved.

### 2.4. Postoperative Evaluation

Patients were examined daily for the first week, weekly for 4 weeks, and monthly thereafter. Ulcer healing time was observed by corneal fluorescein staining during the follow-up. The uncorrected visual acuity (UCVA), intraocular pressure (IOP), and corneal status were recorded. Laser scanning confocal microscopy was applied to determine the extent of local inflammation, and anterior segment optical coherence tomography (AS-OCT) was performed to visualize corneal tissue cicatrization proximal to the ulcer.

### 2.5. Statistical Analysis

The data were analyzed using SPSS® 11.5 software. UCVA before and after surgery was converted to the minimum angle of resolution (log MAR) for calculation purposes and compared with Student's *t*-test. A *P* value of <0.05 was considered to be statistically significant.

## 3. Results

### 3.1. General Information

Eleven patients (11 eyes) with a nonhealing corneal ulcer who underwent the combined surgery were included in the study. Of these, 7 were male and 4 were female, with an age range of 29–68 years (mean ± standard deviation, 58.1 ± 11.4). The etiologies of the corneal ulcers included HSK (8 eyes), corneal graft ulcer (2 eyes), and SJS (1 eye). The ulcers were less than 1/3 of the corneal stroma in depth and 2–5 mm in diameter and lasted for 1–6 months. The site of ulcer in the 11 cases was on the pupillary zone in 5 eyes, on the inferior cornea in 5 eyes, and at the superior cornea in one eye. Combined anterior chamber hypopyon of approximately 1–3 mm was noted in 4 eyes. These patients underwent various medical treatments (antibiotics, antiviral medication, corticosteroids, growth factor, and bandage contact lens) for ≥4 weeks. Of these, 5 eyes received AMT once and 1 eye received AMT twice, but the ulcers remained unhealed (Table 1). Thereafter, active pedicle epithelial flap transposition combined with AMT was performed, and patients were followed-up for 6–24 months (16.9 ± 9.9).

### 3.2. Clinical Examination

**Figure 2:**
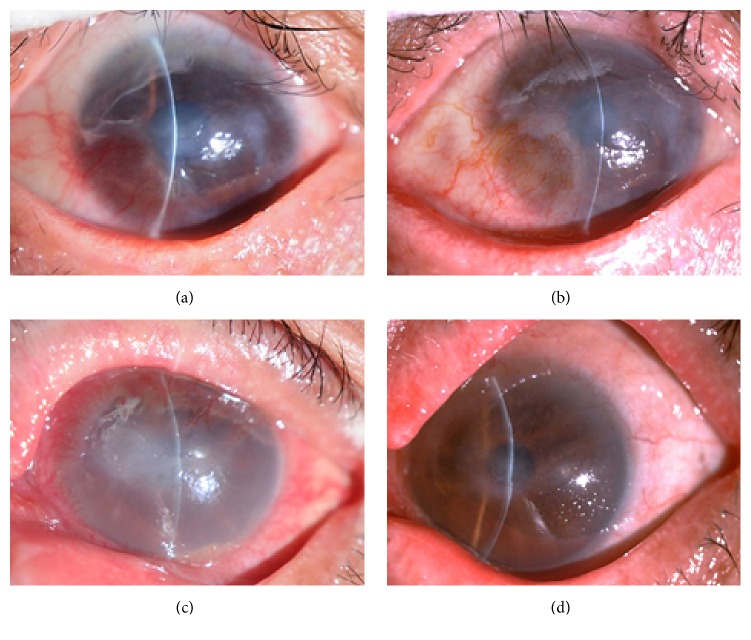
Slit-lamp photographs showing the treatment course of nonhealing corneal ulcers. (a)* Patient 1*: the preoperative uncorrected visual acuity was finger counting. The size of the ulcer was approximately 4.0 × 3.0 mm. The surrounding tissue displayed infiltration and edema. (b) At 1 month after surgery, the corneal ulcer was completely cured, with the relief of corneal opacity, and the postoperative uncorrected visual acuity was 0.1 (1.0 log MAR). (c)* Patient 2*: the preoperative uncorrected visual acuity was hand motion. The ulcer was approximately 5.0 × 2.5 mm. Evident stromal necrosis, edema, and anterior chamber hypopyon were observed. (d) At 1 month after surgery, the corneal ulcer was healed, with the relief of corneal edema, and the postoperative uncorrected visual acuity was 0.1 (1.0 log MAR).

After surgery, all of the epithelial flaps were intact, without displacement or shedding. The corneal ulcers healed from 6 to 15 days (10.8  ±  3.1) postoperatively with negative fluorescein staining. The epithelial defect that resulted from the construction of flap healed within 1-2 days (1.45  ±  0.52). Following surgery, eye hyperemia decreased gradually. The hypopyon observed in 4 patients disappeared at 1-2 weeks. The corneal edema subsided within 2 weeks. The AM patch dissolved between 1 and 3 weeks postoperatively. Corneal opacity was alleviated during the follow-up, with variable effects on visual acuity (VA) (Figure 2). An elevated IOP (30 mmHg) was present in 1 eye and controlled by medication within 3 days. HSK recurrence was observed in 1 patient at 10 weeks and 14 months but without the occurrence of corneal ulcer. Corneal ulcer recurrence was noted in 1 eye (after penetrating keratoplasty) at 3 weeks postoperatively, resulting in a fungal infection and intractable corneal perforation. Finally, the eye was treated successfully with a second penetrating keratoplasty.

### 3.3. Recovery of Visual Acuity

The average preoperative and postoperative UCVA were 1.70 log MAR and 0.82 log MAR, respectively. The percentage of patients with UCVA of <0.05 decreased from 73% (8/11 eyes) preoperatively to 9% (1/11 eyes) postoperatively. The VA increased by at least one row (maximum, six rows) after surgery. The difference in UCVA pre- and postoperatively was statistically significant (*P* = 0.001) (Table 2).

### 3.4. Confocal Microscopy Examination

**Figure 3:**
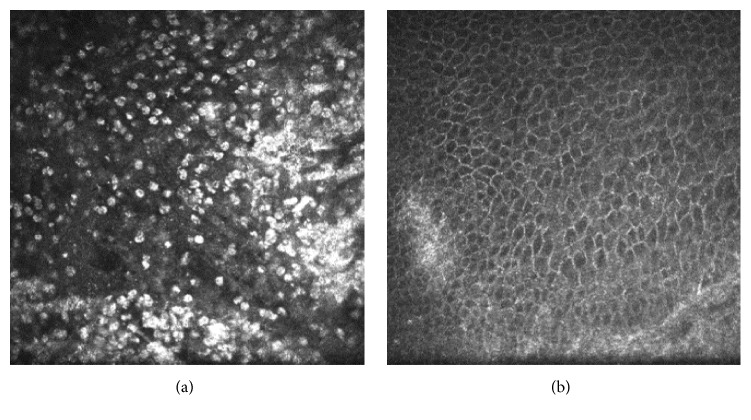
(a) Abundant inflammatory cells were displayed in the epithelium and basal membrane surrounding the epithelial nonhealing region of the ulcer. (b) The number of inflammatory cells decreased significantly at 2 months after surgery.

The inflammatory cells were found to aggregate in the epithelium and basal membrane surrounding the epithelial nonhealing region of the ulcer by confocal microscopy prior to surgery. Inflammatory cell infiltration was noted to be greatly alleviated after surgery (Figure 3).

### 3.5. Anterior Segment Optical Coherence Tomography Examination

**Figure 4:**
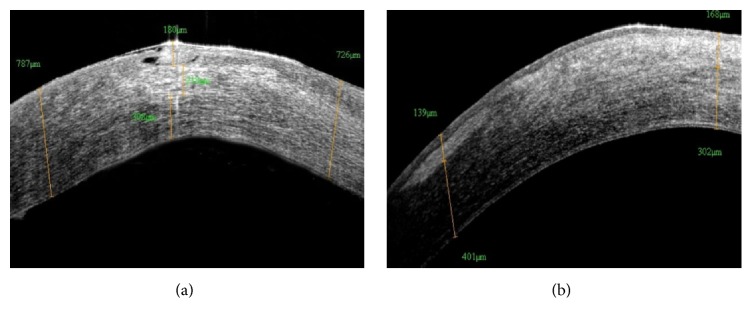
(a) The ulcer was shown to involve approximately 1/3 of the corneal stroma, with stromal edema and infiltration. (b) The combined surgery healed the ulcer, leaving a semitransparent and highly reflective region in the cornea at 2 months postoperatively.

AS-OCT revealed that the surgical treatment had healed the corneal ulcer. Postoperatively, corneal stromal edema decreased, leaving a semitransparent and highly reflective region in the cornea (Figure 4).

## 4. Discussion

The management of nonhealing corneal ulcers is one of the most difficult challenges faced by ophthalmologists because only a few resistant ulcers can be cured solely by medication. Once the nonhealing corneal ulcer tends toward expansion and aggravation, devastating complications can subsequently develop [3]. In the current study, the combination of active pedicle epithelial flap transposition and AMT was found to be effective in promoting ulcer healing and yielding a good cosmesis. Following the treatment, complete ocular surface epithelialization was achieved within an average of 10.8 days.

Initially understanding the underlying reasons for a deferred epithelial healing in this case series was necessary to achieve the most optimal therapeutic effect and recognize the involved mechanisms of the combined surgery in treating resistant corneal ulcers.

The deferred epithelial healing in this study could be attributed to several factors. Firstly, an abnormal or deficient basal membrane caused by corneal melting due to local inflammation hindered epithelial healing, resulting in defective cellular adhesion and recurrent breakdown of the epithelium [10]. Secondly, the inflammatory cells and stromal keratocytes in a distinct, preexisting inflammatory microenvironment restrained epithelial healing by secreting proinflammatory cytokines and proteolytic enzymes [3]. In this study, the primary diseases of corneal ulcer were HSK, corneal graft ulcer, and SJS. It is possible that many of the eyes affected by HSK or after keratoplasty had poor corneal sensation and the eyes with SJS had limbal stem cell pathology. Thus, the conditions of these eyes were predisposed to a deferred epithelial healing.

We believe that the mechanism behind the combined surgery in promoting ulcer healing is related to a comprehensive improvement in the corneal microenvironment through the use of an active epithelial flap. Since the introduction of LASEK to refractive surgery in recent decades, epithelial flaps have successfully been used in subepithelial keratectomy by covering the excimer laser ablation area to reduce inflammation and scarring caused by photorefractive keratectomy [11–13].

It was shown in previous studies that many mediators produced or expressed in the corneal epithelium were effective in regulating inflammatory response and maintaining the homeostasis of the ocular surface, such as Resolvin D1, Resolvin E1, IL1RA, Netrin-1, and UNC5B. These factors reduced the recruitment of inflammatory cells, enhanced phagocytosis, and suppressed the secretion of proinflammatory cytokines [14–18]. It has been demonstrated in previous researches that active epithelial cells can markedly reduce ocular surface inflammation and relieve neurotrophic keratopathy [19–21].

The flap was obtained without the use of alcohol in the current study to preserve the activity of the epithelial flap as far as possible. The active epithelial flap inhibited inflammatory cell infiltration in the inflamed tissue and reduced the quantity of proteinases and cytokines released into the inflammatory cornea. Covering the ulcer with the active epithelial flap provided a relatively healthy substrate and microenvironment. This facilitated epithelial migration, reinforced basal epithelial adhesion, and promoted ocular surface healing. Meanwhile, a decrease in the number of inflammatory cells was detected on confocal microscopy. In addition, thermal cautery was helpful in astringing the melting tissue, gaining better adhesion of the epithelial flap, and ensuring a relative healthy basal membrane. Debridement of the necrotizing tissue around and on the ulcer base helped to improve the corneal microenvironment for ulcer healing. By contrast, a replicating virus and/or severe local inflammatory response could damage the corneal basal membrane and disturb the normal epithelium repair process [8, 22]. Following surgery, the topical administration of corticosteroids further suppresses the immune response, alleviating the stromal edema and improving the visual acuity [23].

The superiority of using the combined surgery over a single AMT treatment was marked. Of all the eyes, 5 eyes received AMT once and 1 eye received AMT twice. However, the corneal ulcers remained unhealed. After the application of a combination of active pedicle epithelial flap transposition and AMT, all of these ulcers were successfully cured within a short time. The results indicate that the epithelial flap might have a greater anti-inflammatory and prohealing effect than that of the amniotic membrane. We speculated that it was the collaborative effect of the epithelial flap and amniotic membrane which inhibited inflammatory cell infiltration and facilitated a relatively healthy microenvironment for ulcer healing. Meanwhile, AMT also ensured epithelial flap adhesion.

Adverse effects were not reported from the use of combined surgery in our study. Its use might also eliminate the need for keratoplasty in some cases. A high risk of immune rejection, epithelial defect, infection, graft melting, and corneal perforation is usually encountered when treating ulcers by corneal transplantation in patients with SJS or other immune diseases [24]. There is less possibility of the immune system being activated and more opportunity of gaining a favorable VA prognosis by employing autogenic epithelial flap transposition in combination with AMT, in addition to reconstructing the corneal surface at an early stage.

In the current study, it was a considerable concern of the clinician whether or not the construction of an epithelial flap would aggravate the primary ocular disease. The epithelial flap was obtained from the transparent cornea close to the limbus for all the eyes because the limbal stem cells are located in this position and taking a flap from here would assure rapid epithelialization. The epithelial defect that resulted from making the flap healed 1.45 days postoperatively and no new region of epithelial defect was identified. Thus, active pedicle epithelial flap transposition combined with AMT was a simple, safe, and effective treatment for nonhealing corneal ulcers.

The treatment of nonhealing sterile corneal ulcers was addressed in the current study but the same approach could not be expected to be effective in patients with recalcitrant corneal ulcers associated with active fungal or bacterial infection. Furthermore, the optimal time for this procedure remains unknown. It is unknown whether the method should be delayed until all conventional treatment has failed or instead be considered earlier. Although the outcome of combined surgery was good in the current study, a randomized, controlled study with a larger sample size is needed for further investigations.

## 5. Conclusion

The combination of active pedicle epithelial flap transposition and AMT can reduce inflammatory response, promote epithelial healing, and restore useful vision in cases of nonhealing corneal ulcers.

## Figures and Tables

**Table 1:** Prior treatment for nonhealing corneal ulcers.

Etiology	Course (month)	Treatment
HSK	4	Medication and AMT
HSK	2.5	Medication and AMT
HSK	2	Medication and AMT
HSK	1	Medication and AMT
HSK	1	Medication and AMT
HSK	1.5	Medication
HSK	1	Medication
HSK	1	Medication
Corneal graft ulcer	1	Medication
Corneal graft ulcer	1	Medication and AMT
SJS	6	Medication

HSK: herpes simplex keratitis; SJS: Stevens-Johnson syndrome; AMT: amniotic membrane transplantation.

**Table 2:** The difference in uncorrected visual acuity (log MAR) before and after surgery.

Difference of UCVA					
Mean	SD	*N*	*t*	*P* value^a^	95% CI
0.88	0.60	11	4.91	0.001	0.48–1.28

UCVA: uncorrected visual acuity; SD: standard deviation; *N*: number of eyes; CI: confidence interval.

^a^Paired *t*-test.
